# Multiple Environmental Factors Shaping Hopanoid-Producing Microbes Across Different Ecosystems

**DOI:** 10.3390/microorganisms13061250

**Published:** 2025-05-28

**Authors:** Ruicheng Wang, Zhiqin Xi, Linfeng Gong, Han Zhu, Xing Xiang, Baiying Man, Renju Liu, Zongze Shao, Hongmei Wang

**Affiliations:** 1 Key Laboratory of Marine Genetic Resources, Third Institute of Oceanography, Ministry of Natural Resources, Xiamen 361005, China; gonglinfeng260592@163.com (L.G.);; 2 State Key Laboratory of Biogeology and Environmental Geology, China University of Geosciences, Wuhan 430078, China; 3 College of Pharmacy, Nankai University, Tianjin 300071, China; 4 College of Life Science, Shangrao Normal University, Shangrao 334001, China

**Keywords:** hopanoid-producing microbes, *sqhC*, multiple factors, various ecosystems

## Abstract

Hopanoids are a series of important lipid biomarkers in the bacterial cellular membranes that are found ubiquitously in different spatial and temporal environments. Squalene-hopane cyclase, a key and prerequisite molecular component of the hopanoid biosynthesis pathway, is encoded by the *sqhC* gene. To investigate the composition, niche, and distribution of microbial *sqhC*-containing communities, we analyzed hopanoid producer data and environmental parameters across different ecosystems on the basis of sequencing reads of peat samples from increasing gradient depths across peatland profile C in the Dajiuhu Peatland, as well as data collected from available published papers. The results indicated that the acidic Dajiuhu Peatland harbored mainly Acidobacteria (59.16%) among its *sqhC*-containing groups. The main composition of hopanoid producers in the peatland was different from that in other ecosystems, with Alphaproteobacteria found in soil (37.78%), cave (48.21%), hypersaline lagoon (34.04%), and marine (32.59%) ecosystems; Betaproteobacteria, Gammaproteobacteria, and Deltaproteobacteria found in reef (100%), acid mine drainage (55.00%), and estuary, mangrove, and harbor (39.66%) ecosystems; and an unknown cluster found in freshwater (29.43%) and hot spring (89.58%) ecosystems. Compared with other phyla or sub-phyla, Alphaproteobacteria, Betaproteobacteria, and Gammaproteobacteria were the most widespread, occurring in eight ecosystems. Peatland was significantly separated from the other nine ecosystem modules in the occurrence network, and the marine ecosystem had the greatest impact on the eco-network of *sqhC* microbes. An RDA indicated that pH, DO, salinity, and TOC had significant impacts on *sqhC*-containing microbial communities across the different ecosystems. Our results will be helpful to understanding the diversity, composition, and distribution of the *sqhC* community and its response to multiple environmental factors across different ecosystems.

## 1. Introduction

Hopanoids are a series of pentacyclic triterpenoid lipid compounds from bacterial cellular membranes and are found ubiquitously in diverse modern environments, as well as geological history samples [1]. Because of their good preservation over extremely long times, they are normally considered as “molecular fossils” for reconstructing geological records [2,3]. Hopanoids also play a key role in the microbial carbon pump (MCP), named the hopanoid MCP, with a special rich-C structure across spatial and temporal scales. Hopanoid biosynthesis is catalyzed by squalene-hopene cyclase, encoded by the *sqhC* gene, cyclizing linear squalene into diploptene and/or diplopterol for adaptation to environmental stress [4]. It is normally found in ~5–10% of terrestrial and marine bacteria in various environments [5]. Among these special microbes, those with the hopanoid MCP are one of the most significant and important groups providing microbial carbon storage in the global carbon cycle, and they efficiently drive almost unidirectional carbon flux toward recalcitrant dissolved organic carbon [6]. Different hopanoid compounds from hopanoid producers might respond to different environments and be produced by different communities at different metabolic rates [7]. Thus, the hopanoid-producing microbes might have a significant impact on the eco-distributions and metabolic rates of hopanoid production in various environments. Understanding their diversity, composition, and distribution in different habitats, along with their response to environmental changes, will help us to decipher the hopanoid producers and associated hopanoids in the global carbon store.

To elucidate the diversity, composition, and distribution of hopanoid producers in natural conditions, the functional gene *sqhC* (encoding squalene-hopene cyclase) responsible for the cyclization of squalene—the first step in hopanoid synthesis, resulting in the formation of the distinctive polycyclic hopanoid skeleton—is proposed [8]. The *sqhC* gene has been one of the genes most widely used to study hopanoid-producing bacteria and their phylogeny in natural environments. On the basis of clone sequencing and metagenomics, *sqhC*-containing bacteria have been observed in various environments, and the dominators have changed significantly. For example, cave ecosystems are dominated by *sqhC* sequences from Alphaproteobacteria and Gammaproteobacteria [9]. Estuary, mangrove, and harbor environments appear to contain relatively more sequences from Alphaproteobacteria, Deltaproteobacteria, Acidobacteria, and Planctomycetes [10]. Moreover, Verrucomicrobia and Ascomycota are found only in marine and peatland environments, respectively [11]. In addition to environmental factors, a previous study showed that pH is a main factor controlling *sqhC*-containing microbes among cave, peatland, and acid mine drainage ecosystems [12], as well as the abundance and components of hopanoids in pure cultures of *Rhodopseudomonas palustris* TIE-1 strains [13]. Moreover, the yield of bacteriohopanepolyol (BHPs) and configuration of hopanoids from hopanoid producers are influenced and regulated by the pH level in acidic peat [14]. Nitrospinae appears to be the dominant hopanoid group in low-oxygen areas, and it couples with the nitrogen cycle in marine environments and suggests that the concentrations of oxygen and nitrite have an important impact on the community structure and physiological metabolism of hopanoid producer groups in low-oxygen marine areas [15]. Nevertheless, the composition and distribution of hopanoid producers in various kinds of ecosystems, along with the relationship between hopanoid producer groups and environmental factors on the global scale, remain largely unknown.

**Figure 1:**
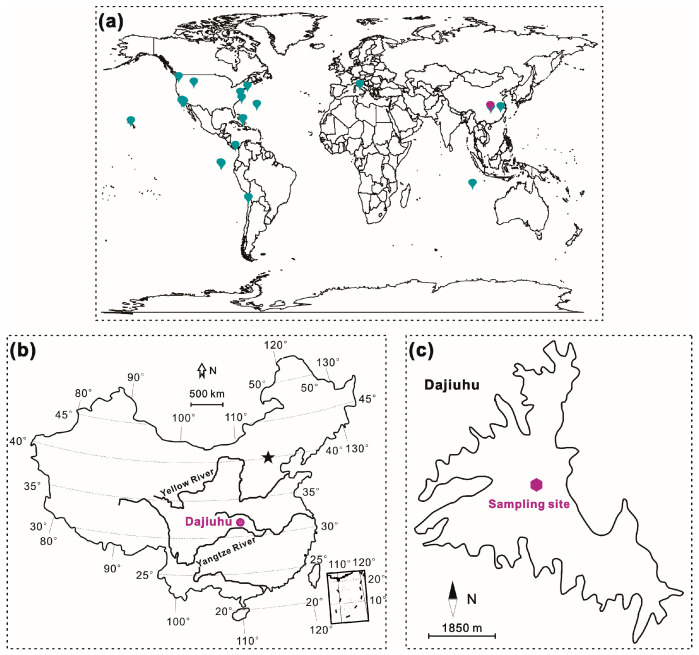
The distribution of the study sites: (**a**) the study sites of the available sequence data, displayed on a world map with collecting data of dark moderate cyan and sequencing data of medium dark magenta; (**b**) the location of the Dajiuhu Peatland and the star standing for Beijing; (**c**) the peat sampling site.

To investigate hopanoid producers in natural environments and their correlation with environmental factors, peat samples were collected from a peatland profile in the Dajiuhu Peatland (Figure 1b). Additionally, all available *sqhC* gene sequence data and community compositions at the phylum or sub-phylum level (Supplementary Materials, Table S1) up to the end of 2024 were obtained from the literature. A phylogenic and statistical analysis was conducted on the sample and literature data to elucidate the relationships between *sqhC*-containing bacteria and environmental factors such as pH, total organic carbon (TOC), salinity, dissolved oxygen (DO), and temperature (T). Our data will expand our knowledge about the diversity and natural distribution of hopanoid-producing bacteria and the environmental factors shaping their geographical distribution across different ecosystems.

## 2. Materials and Methods

### 2.1. Study Site Description

The Dajiuhu Peatland (31°28′50″ N, 110°00′09″ E) is a closed subalpine basin located in the middle reaches of the Yangtze River, central China. The modern dominant peat-forming plants include sedge species, *S*. *officinalis*, and *Sphagnum palustre* [16]. The basin has a mean elevation of 1730 m and a total area of 16 km^2^. The climate in this region is dominant by the East Asian summer monsoon, with hot-wet summers coupled with cold-dry winters, mean annual precipitation of 1560 mm, and a mean annual temperature of 7.2 °C [17,18].

### 2.2. Sampling

Sediment samples were collected from profile C (PC) in the Dajiuhu Peatland. All the utensils and tools used for sampling were sterilized. An open peat profile was dug at the Dajiuhu Peatland, and four sediment samples were collected at each sampling depth (5 cm, 25 cm, 55 cm, and 105 cm). The samples were designated PC5, PC25, PC55, and PC105 according to their sampling depth from the surface. Each sample was sampled from the bottom to the surface using aseptic zip-bags and immediately stored on dry ice. All peat samples were taken back to the Geomicrobiology Lab and stored at −80 °C.

### 2.3. Physicochemical Analysis

The dissolved oxygen (DO), pH, and temperature (T) were measured in situ with an HQ40d Portable Meter (HACH, Loveland, CO, USA) [19]. The total organic carbon (TOC) content and salinity were analyzed with a DR2800 Portable Spectrophotometer (HACH, Loveland, CO, USA) [20].

### 2.4. Genomic DNA Extraction and Functional Gene Sequencing

The genomic DNA was extracted from 0.5 g of dry peat sample powder with a FastDNA Spin Kit for Soil (MP, Irvine, CA, USA). The DNA concentration and purity were measured with a Nanodrop ND-2000 spectrophotometer (Thermo Fisher Scientific, Waltham, MA, USA). Sequencing libraries were generated using a NEBNext^®^ Ultra^TM^ DNA Library Prep Kit for Illumina (NEB, Beverley, MA, USA) following the manufacturer’s recommendations, and index codes were added to the attribute sequences of each sample. One cluster of index-coded samples was processed on a cBot Cluster Generation System using TruSeq PE Cluster Kit v3-cBot-HS (Illumina, San Diego, CA, USA) according to the manufacturer’s instructions. After cluster generation, the libraries were sequenced on an Illumina HiSeq 2500 platform, and paired-end reads were generated.

### 2.5. Gene Reads Analysis

Raw reads were processed to obtain high-quality reads according to the following stringent filtering standards: (1) removing reads with ≥10% unidentified nucleotides (N); (2) removing reads with >50% bases with phred quality scores of ≤20; and (3) removing sequences aligned to the barcode adapter.

The clean sequencing data for all samples were assembled individually by using MEGAHIT (version 1.1.3) to generate sample-derived assemblies within stepping over a k-mer range of 21 to 99; combinations of paired or singleton reads were realigned with the sequence assembly using BWA to obtain the overall de novo assembly statistics [21,22]. Unmapped reads from the samples were pooled for re-assembly to generate a mixed assembly. Then, the final assembly obtained was used for further analysis.

Open reading frames (ORFs) were predicted on the basis of the contigs > 500 bp using MetaGeneMark 31 (version 3.26) [23]. Those of length ≥300 bp chosen from the sample data were pooled and combined based on ≥95% identity and >90% coverage using CD-HIT in order to exclude redundant genes for the downstream step [24]. These reads were re-aligned to annotated genes using BWA with a read count of >2. Unique ORFs were annotated by using DIAMOND (version 0.9.9) within KEGG, CAZY, and eggNOG [25].

### 2.6. Phylogenetic Analysis

The updated reference sequences of the *sqhC* gene from the NCBI database were pooled with our sequences for an alignment analysis. All *sqhC* sequences were firstly translated into partial protein sequences using MEGA 6.0 and then aligned by using the ClustalW algorithm within conserved motifs of amino acid sequences among the SHCs from all nearby hopanoid phyla [26,27,28]. A phylogenetic tree was constructed via bootstrapping with 1000 iterations using the neighbor-joining p-distance method. The original *sqhC* sequencing reads from this research are included in the Supplementary Materials, Table S6.

### 2.7. Statistical Analysis

The diversity indices of hopanoid producer groups in the Dajiuhu Peatland were calculated at the OUT level by using Mothur with a cutoff of 5% evolutionary distance [29]. The composition and structure of hopanoid producer communities in different ecosystems were analyzed and compared at the phylum or sub-phylum level on the basis of the phylogenetic tree and known data from reports published prior to the end of 2024. The microbial species occurrence distribution patterns of the hopanoid producers were based upon the hopanoid-producing microbial distributions across different ecosystems. To elucidate the relationship between microbial *sqhC* communities and environmental parameters, a statistical analysis was conducted on all the available *sqhC* data and physical-chemical factors by using Canoco (version 5.0) with the redundancy analysis (RDA) method [30]. The significant parameters for each variable were calculated using unrestricted-permutation Monte Carlo tests for the *p*-value and *F*-ratio [31].

The correlation network was generated from the distribution matrix of hopanoid-producing microbes belonging to various habitats by using R version 4.4.3 coupled with Gephi version 0.9.7 with the standard parameter setting [32]. The matrix contained the relative abundances of hopanoid producers across all samples between phylum-level species classifications and eco-distribution sites. Pearson correlation coefficients (*r* > 0.6 or *r* < −0.6) coupled with a significance of *p* < 0.001 were all integrated into the eco-network analysis. Each node represented one eco-distribution site, and each edge represented a correlation between two nodes of the network. The network was characterized by topology indices, including the average path length, average degree, graph density, network diameter, clustering coefficient, and modularity. Node-level topological parameters such as the degree, betweenness, eigenvector centrality, and closeness were also calculated.

## 3. Results

### 3.1. Diversity and Composition of Hopanoid-Producing Microbe Communities in the Dajiuhu Peatland

The Chao1 estimator, coverage estimator (ACE), Shannon–Wiener index, observed richness (Sobs), and Simpson index (1/D) were employed as community richness indices. Significant variances were observed among the four peat samples for hopanoid producer groups across the PC samples (Table 1). The Chao1, ACE, Sobs, Shannon, and Simpson (1/D) values for PC25 were higher than those for other depths, and the minimum values were observed for the PC55 sample. The diversity indices for surface sample PC5 were greater than those for subsurface sample PC105, suggesting that upper peat samples (PC5 and PC25) had greater diversity and richness than lower samples (PC55 and PC105).

**Figure 2:**
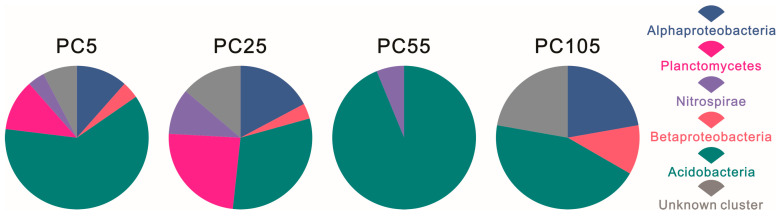
Composition of hopanoid-producing microbe communities in PC samples from Dajiuhu Peatland.

Regarding the compositions of the hopanoid-producing microbe communities in Figure 2, Acidobacteria was the dominant phylum in all four samples PC5, PC25, PC55, and PC105, with relative abundances of 61.54%, 31.03%, 93.75%, and 44.44%, respectively. Besides this, Alphaproteobacteria, Betaproteobacteria, and an unknown cluster were found only in PC5, PC25, and PC105, with the highest abundance in PC105 at percentages of 22.22%, 11.11%, and 22.22%, respectively. Planctomycetes and Nitrospirae were not found in the PC55 and PC105 samples, but higher abundance (24.14% and 10.34%) was observed in the PC25 sample.

### 3.2. Composition of Hopanoid-Producing Communities Across Different Ecosystems

**Figure 3:**
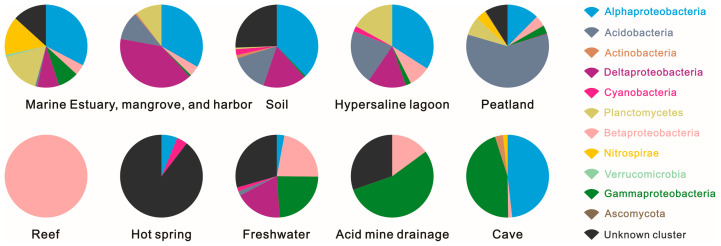
Composition of hopanoid-producing groups at the phylum or sub-phylum level across different ecosystems.

Acidobacteria dominated in the peatland ecosystem, contributing 59.16% of the hopanoid-producing microbes. In soil; hypersaline lagoon; and estuary, mangrove, and harbor ecosystems, the same phylum contributed more than 10% of hopanoid-producing microbes. Alongside Acidobacteria, Alphaproteobacteria was another main group (12.45%) in the peatland, followed by an unknown cluster (8.92%) and Planctomycetes (7.22%). Alphaproteobacteria also dominated in soil (37.78%); cave (48.21%); hypersaline lagoon (34.04%); estuary, mangrove, and harbor (33.28%); and marine (32.59%) environments. In contrast to Alphaproteobacteria, Betaproteobacteria mainly contributed over 10% of hopanoid producers in acid mine drainage, reef, and freshwater environments, followed by hypersaline lagoons (8.51%). The freshwater ecosystem also contained Gammaproteobacteria (23.75%) and Deltaproteobacteria (18.39%). On the other hand, Gammaproteobacteria dominated cave (45.31%) and acid mine drainage (55.00%) ecosystems, while Deltaproteobacteria dominated soil (16.88%); hypersaline lagoon (14.89%); and estuary, mangrove, and harbor (39.66%) ecosystems in Figure 3.

**Figure 4:**
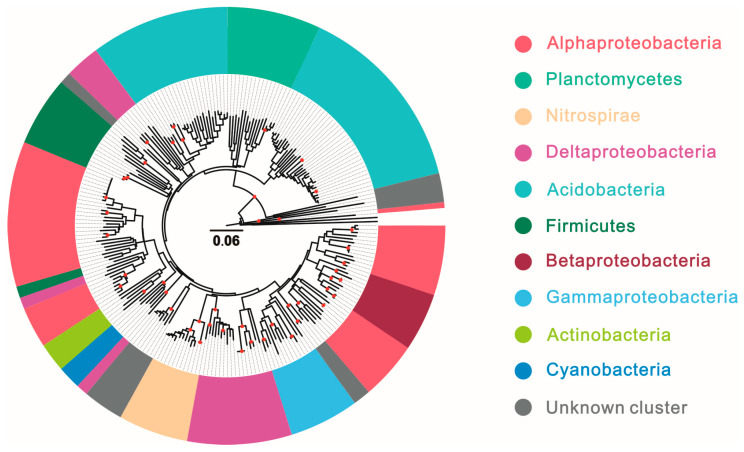
Phylogenetic tree based on *sqhC* sequences of hopanoid producers across different ecosystems; solid red circles on the phylogenetic tree highlight tree nodes with bootstrap values of ≥90%.

Planctomycetes showed in Figure 4 played an important role among the hopanoid-producing microbes and dominated with relative abundances of more than 10% in hypersaline lagoon (17.02%); estuary, mangrove, and harbor (10.33%); and marine (16.46%) ecosystems, followed by peatland (7.22%). Nitrospirae dominated the marine ecosystem with a percentage of 15.05% of the total hopanoid producers. Lastly, unknown cluster microbes are potential and unclear hopanoid producers and occupied higher percentages in various environments, such as soil (25.55%), acid mine drainage (30.50%), freshwater (29.43%), marine (13.29%), and hot spring (89.58%) ecosystems.

### 3.3. Species Occurrence Patterns of Hopanoid Producers Among Different Ecosystems

**Figure 5:**
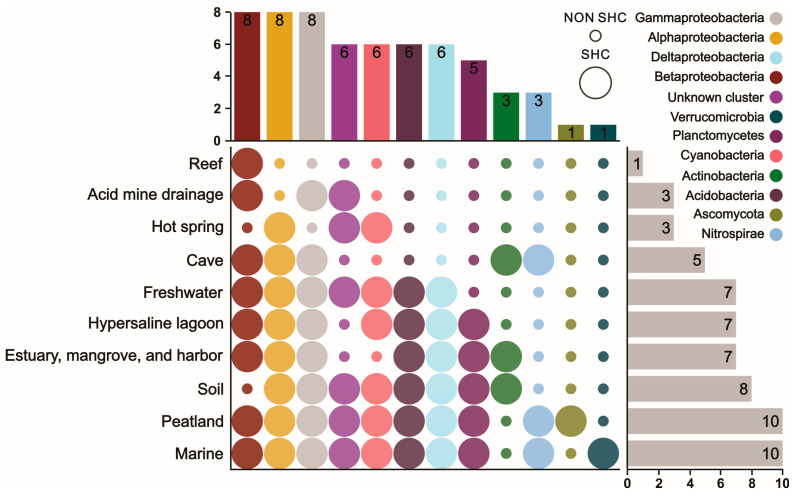
Species occurrence patterns of hopanoid producers among different ecosystems.

In detail, a total of 12 groups were distributed in 10 different ecosystems. Among all the different ecosystems (Figure 5), peatland and marine were the richest in microbial species (10), while the reef ecosystem had only Betaproteobacteria. Other environmental conditions, such as acid mine drainage and hot spring, were associated with three hopanoid producer groups. Besides these, cave; hypersaline lagoon; estuary, mangrove, and harbor; freshwater; and soil environments contained five, seven, seven, seven, and eight hopanoid groups, respectively.

On the other hand, microbes from Alphaproteobacteria, Betaproteobacteria, and Gammaproteobacteria were the most widely distributed and survived in 8 of the 10 ecosystems. Deltaproteobacteria, the unknown cluster, Cyanobacteria, and Acidobacteria were distributed in six kinds of different ecosystems. The least prevalent groups, Verrucomicrobia and Ascomycota, survived in only 1 of the 10 ecosystems.

### 3.4. Correlations Between Hopanoid-Producing Groups and Environmental Factors Across Different Ecosystems

**Figure 6:**
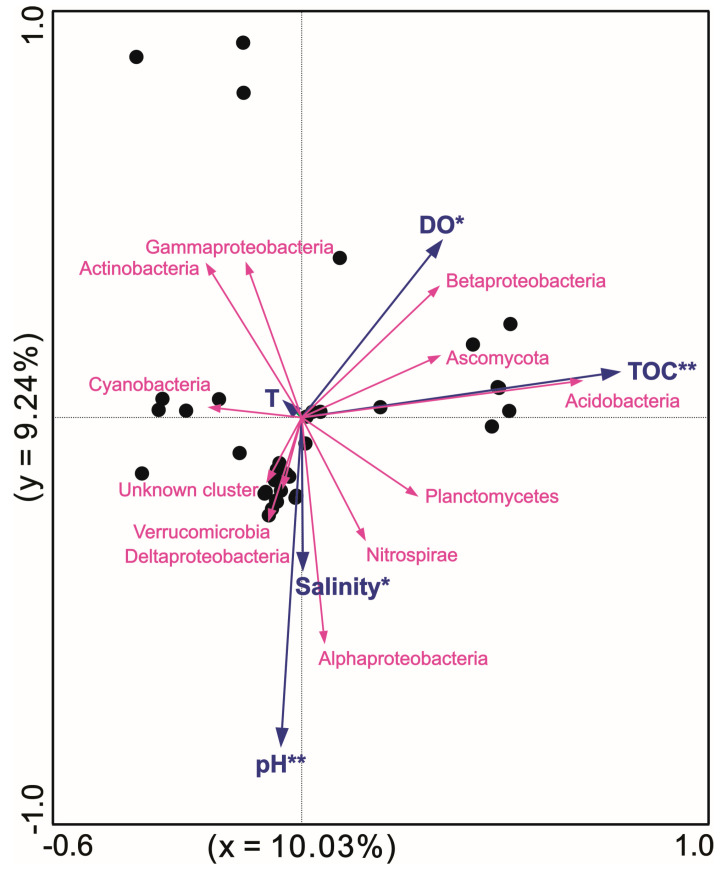
RDA between *sqhC*-containing microbes and physiochemical parameters at different sites with * and ** standing for significant *p* < 0.05 and extremely significant *p* < 0.01 respectively.

An RDA was conducted to analyze the effect of environmental factors on hopanoid producers (Figure 6). Generally, the clones and metagenomic data from the literature, along with our sequences, fell into 12 phyla adaptive to these multiple environmental parameters. Acidobacteria was positively correlated with the TOC content; however, Cyanobacteria should be negatively correlated with the TOC content. In detail, the factors pH (*p* = 0.002, *F* = 3.1) and TOC (*p* = 0.006, *F* = 3.0) highly significantly influenced the hopanoid producer microbes, while salinity (*p* = 0.02, *F* = 2.5) and DO (*p* = 0.034, *F* = 2.4) significantly shaped the hopanoid producers. Overall, the TOC, pH, salinity, DO, and T explained 8.8%, 8.4%, 6.6%, 6.1%, and 1.8% of the whole hopanoid producer groups, respectively. In total, 19.28% of the variance in the hopanoid producers and environmental parameters was explained by two axes.

### 3.5. Occurrence Network of Distribution of Hopanoid-Producing Microbes Among Various Ecosystems

**Figure 7:**
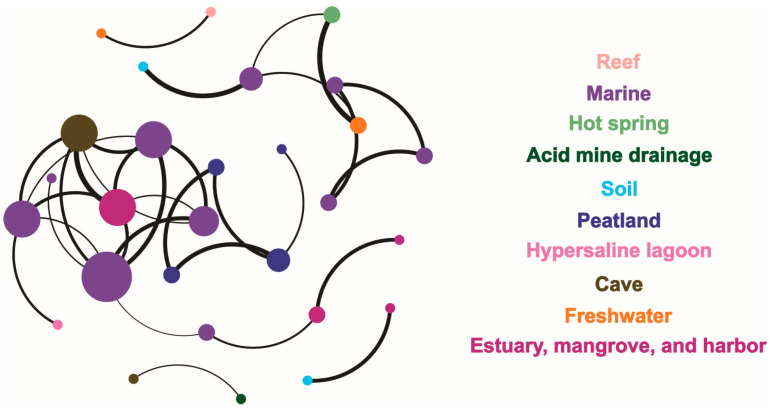
A network correlative analysis of the eco-distribution of hopanoid producers in the different ecosystems.

The network relationship of the sites of hopanoid producers was calculated with the data sites belonging to various eco-modules across the different ecosystems. In total, 28 nodes and 33 edges were positively correlated (Figure 7). The marine module had the greatest impact on the network structure. Specifically, the average degree, network diameter, network density, clustering coefficient, average weighted degree, and modularity were 2.36, 4.39, 0.09, 0.7, 2.15, and 0.68, respectively.

The total network based on modularity fell into 10 modules in Figure 7. Besides the greatest—the marine module—the peatland; cave; freshwater; and estuary, mangrove, and harbor modules occupied greater parts of the network. According to its relative values, the marine module was related to almost all of the modules, excluding peatland, acid mine drainage, and reef. The cave system module was closer to the modules for marine; acid mine drainage; and estuary, mangrove, and harbor. The peatland module indicated an independent ecosystem and had no relationship with the other nine modules. The freshwater module was closer to the reef, hot spring, and marine modules, while the estuary, mangrove, and harbor module shared occurrence relationships with the cave, marine, and soil ecosystems. The reef and acid mine drainage modules were each related with only one module: freshwater and cave, respectively. The hot spring module was closer to the two modules for marine and freshwater. The soil module was also closer to two modules: marine and estuary, mangrove, and harbor. The hypersaline lagoon presented a relationship in the occurrence network with only the marine ecosystem. In all, the relationship values between different nodes among the different ecosystem modules showed average *r* and *p* values of 0.91 and 0.00016, respectively.

## 4. Discussion

### 4.1. Characteristics of Hopanoid Producers from Profile C in Dajiuhu Peatland and Differences in Hopanoid Producers Between Peatland and Other Ecosystems

Acidobacteria, the most dominant *sqhC*-containing community at the phylum or sub-phylum level from profile C in the Dajiuhu Peatland, has also been reported in other environments previously, but it varies greatly in its relative abundance with respect to *sqhC* microbes. For example, Acidobacteria contributed only 10–21% to the abundance of hopanoid producers in samples from freshwater; soil; marine; hypersaline lagoon; and estuary, mangrove, and harbor ecosystems but about 60% to their abundance in the Dajiuhu Peatland. Consistent with the network analysis, the peatland ecosystem showed independence from the other nine modules, within a significant impact on the network. This might have been caused by a high abundance of Acidobacteria relative to the total amount of hopanoid producers in the peatland. Studies have shown that Acidobacteria microbes are well adapted to low-nutrient and acidic survival conditions [33,34]. Considering the low pH and oligotrophic conditions in the Dajiuhu Peatland, providing favorable environmental conditions for Acidobacteria, it is highly reasonable that Acidobacteria is dominant in the Dajiuhu Peatland [35,36].

Compared with Betaproteobacteria, Gammaproteobacteria, and Deltaproteobacteria, Alphaproteobacteria accounted for greater relative abundance in the *sqhC*-containing community (12.45%) in the Dajiuhu Peatland. Moreover, the Alphaproteobacteria community was higher in abundance than the Betaproteobacteria, Gammaproteobacteria, and Deltaproteobacteria communities in soil, cave, hypersaline lagoon, marine, and hot spring ecosystems.

It is further notable that Planctomycetes was detected in soil; marine; hypersaline lagoon; and estuary, mangrove, and harbor ecosystems with percentages of less than 18%, reaching as low as 0.63% and 7.22% in soil and the Dajiuhu Peatland. Actually, Planctomycetes favors a biofilm lifestyle, adhering to surfaces in aquatic conditions, such as in marine, estuary, seaweed, and harbor ecosystems [37]. Nitrospirae contributed 4.09% of hopanoid producer microbes in the Dajiuhu Peatland, less than in the marine ecosystem (15.05%). The Dajiuhu Peatland is also a typical *Sphagnum*-dominated and oligotrophic bog [38]. Nitrogen nutrition highly depends on atmospheric nitrogen deposition by means of microbial nitrogen fixation [39]. Nitrospirae, a key participant in the nitrification process, has been confirmed to be capable of oxidizing ammonia to nitrite and then to nitrate, thus contributing to nitrogen nutrition in the Dajiuhu Peatland [40] and having higher relative community abundance.

An unknown cluster accounted for 8.92% of hopanoid producers and was also found in soil, acid mine drainage, freshwater, marine, and hot spring ecosystems with an abundance of more than 10% of the total *sqhC*-containing microbes. In particular, the acid mine drainage and hot spring ecosystems harboring a high abundance of unknown hopanoid producers suggests an important metabolic mechanism and function of the *sqhC* gene in defense against extreme conditions [41,42].

### 4.2. Multiple Environmental Factors Shaping Hopanoid-Producing Microbes Across These Various Ecosystems

Combined with all the *sqhC* data reported in the literature, an RDA showed that pH, TOC, DO, and salinity are important environmental factors significantly shaping the composition of *sqhC*-containing communities in natural environments. The pH is confirmed to shape the geographical distribution of hopanoid producers, as well as the community composition in soils on a large scale [12]. Moreover, studies have suggested that hopanoids may play an indirect role in pH homeostasis, with certain hopanoid derivatives being of particular importance [43]. Thus, pH gradients may regulate the physiological metabolism and community composition of hopanoid producers. This also agrees with the network distribution of the low-pH peatland and acid mine drainage modules being far from the other modules. Besides pH, TOC should be another key and significant factor influencing the *sqhC*-containing community. High TOC seems to favor a higher OTU number for the *sqhC* gene, and that suggests a heterotrophic lifestyle of hopanoid producers [44]. Microbial hopanes have been found to be ^13^C-enriched and show comparable values of δ^13^C to their organic carbon sources, such as special higher plants, aerobic heterotroph microbes, and other labile ^13^C-enriched carbon compounds (possibly carbohydrates) [45,46]. The correlation between microbial maximum growth rates and initial TOC contents suggests that rich TOC promotes microbial growth and cellular metabolism, such as in the *Sphingomonas yanoikuyae* JAR02 strain [47].

Besides pH and TOC, two other parameters, DO and salinity, can also significantly influence hopanoid producers. As we know, the hopanoid fluxes of *sqhC*-containing bacteria indicating hydrological variations confirm that the dominant contribution of hopanoid producers to hopanoids is made by aerobes in peatland [3]. Furthermore, several studies have indicated that the BHP concentration and structural diversity both increase as the O_2_ concentration decreases, and some BHP structures are specific to bacterial groups in low-O_2_ marine zones [48,49]. Thus, the oxygen concentration is also an important influencing factor for hopanoid producers, affecting their bacterial community structure, metabolism, and abundance. Lastly, salinity may be a potential and key environmental factor impacting hopanoid producer groups. The 2-methyhopanoids detected in marine sediments are largely interpreted as biomarkers for Cyanobacteria because of their frequent occurrence in cultured Cyanobacteria [50]. However, the discovery of their presence among a wide range of soil bacteria, including Rhizobiales and Actinobacteria [51,52,53], as well as a strong association of 2-methyhopanoids with modern terrigenous environments [54,55], suggest that 2-methyhopanoids reflect different microbial information outside of marine environments. Microbial organic profiles obtained from hopanoid producers of 2-methyhopanoid compounds indicate that different microbial ecosystems have adapted to different geochemical environments characterized by salinity and oxygen levels [56]. This adaption to salinity might have been caused by the Cryogenian period in geological history [57,58] and appears to be consistent with the hypersaline lagoon and marine modules in the occurrence network.

Besides the above environmental factors, there may be other factors impacting hopanoid-producing bacteria, such as temperature. However, temperature is not significant as pH, TOC, DO, or salinity. With greater spatial and temporal scales, sample reads and environmental parameters might provide more details regarding the diversity, composition, and distribution of hopanoid producers in various habitats and metabolic mechanisms against stress conditions. This will be helpful in realizing the hopanoid MCP in the global carbon cycle.

## 5. Conclusions

Different *sqhC*-containing communities were obvious at different depths of profile C of the Dajiuhu Peatland, and Acidobacteria was dominant among the *sqhC*-containing microbes. Additionally, the *sqhC*-containing microbes in the peatland were obviously different from those found in other ecosystems, such as Alphaproteobacteria in soil, cave, hypersaline lagoon, and marine ecosystems; Betaproteobacteria, Gammaproteobacteria, and Deltaproteobacteria in reef, acid mine drainage, and estuary, mangrove, and harbor ecosystems, respectively; and unknown clusters in freshwater and hot spring ecosystems.

Moreover, classes of Proteobacteria, consisting of Alphaproteobacteria, Betaproteobacteria, and Gammaproteobacteria, were the most widespread in eight ecosystems at the phylum and sub-phylum level. The network relationship showed that the marine module had the greatest impact on the hopanoid-producing microbes, and the peatland module indicated an independent ecosystem of hopanoid producers far from other modules in the occurrence network. A statistical RDA indicated that pH, DO, salinity, and TOC could significantly impact *sqhC*-containing microbial communities across the different ecosystems. Our results will be helpful to understanding the structure and distribution of *sqhC* microbial groups and the response to multiple environmental factors among these different ecosystems.

## Figures and Tables

**Table 1:** Diversity and richness of hopanoid producer groups in profile C of Dajiuhu Peatland.

Sample	Sobs	ACE	Chao1	Shannon	Simpson (1/D)
PC5	26	125.67	89.25	3.22	135.34
PC25	27	378	189.5	3.28	377.93
PC55	8	11.8	9.5	1.95	10.11
PC105	8	36	18.5	2.04	36

## Data Availability

The original contributions presented in this study are included in the article/Supplementary Material. Further inquiries can be directed to the corresponding authors.

## Supplementary Materials

The following supporting information can be downloaded at https://www.mdpi.com/article/10.3390/microorganisms13061250/s1. Table S1: Available data of compositions of hopanoid producers until the end of 2024. Table S2: Environmental parameters of study sites among the various ecosystems. Table S3: Study sites belonging to the various ecosystems. Table S4: Correlation and significant values of network. Table S5: Parameter values of network. Table S6: Original sequencing reads of this study in the Dajiuhu Peatland.
